# Improving Attitude Estimation Using Gaussian-Process-Regression-Based Magnetic Field Maps

**DOI:** 10.3390/s21196351

**Published:** 2021-09-23

**Authors:** Prince E. Kuevor, James W. Cutler, Ella M. Atkins

**Affiliations:** 1 Robotics Institute, University of Michigan, Ann Arbor, MI 48109, USA; 2 Aerospace Engineering, University of Michigan, Ann Arbor, MI 48109, USA; jwcutler@umich.edu (J.W.C.); ematkins@umich.edu (E.M.A.)

**Keywords:** Gaussian processes, magnetic field mapping, Kalman filter, attitude estimation, unmanned aerial vehicle (UAV)

## Abstract

Magnetometers measure the local magnetic field and are present in most modern inertial measurement units (IMUs). Readings from magnetometers are used to identify Earth’s Magnetic North outdoors, but are often ignored during indoor experiments since the magnetic field does not behave how most expect. This paper presents methods to create, validate, and visualize three-dimensional magnetic field maps to expand the use of magnetic fields as a sensing modality for navigation. The utility of these maps is measured in their ability to accurately represent the magnetic field and to enable dynamic attitude estimation. In experiments with motion capture truth data, a small multicopter with three-axis inertial measurements, including magnetometer, traversed five flight profiles distinctly exciting roll, pitch, and yaw motion to provide interesting trajectories for attitude estimation. Indoor experimental results were compared to those outdoors to emphasize how spatial variation in the magnetic field drives the need for our mapping techniques. Our work presents a new way of visualizing 3D magnetic fields, which allows users to better reason about the magnetic field in their workspace. Next, we show that magnetic field maps generated from coverage patterns are generally more accurate, but training such maps using observations from desired flight paths is sufficient in the vicinity of these paths. All training sets were interpolated using Gaussian process regression (GPR), which yielded maps with <1 μT of error when interpolating between and extrapolating outside of observed locations. Finally, we validated the utility of our GPR-based maps in enabling attitude estimates in regions of high magnetic field spatial variation with experimental data.

## 1. Introduction

Unmanned aerial vehicles (UAVs) are being used for a number of different mission designs including surveillance and package delivery. Such tasks are more tractable on vehicles with some degree of autonomy that can handle navigation and control on their own. For applications outside of buildings and urban canyons, vehicles can use the Global Positioning System (GPS) for positioning and leverage maps of Earth’s magnetic field such as the World Magnetic Model (WMM) to observe vehicle heading and estimate attitude. However, indoor environments have no such magnetic field maps to help with attitude estimation and lose the ability to use GPS for their position.

Several sensor modalities such as vision and wireless ranging can be leveraged for indoor navigation [1,2,3]. However, these modalities can be sensitive to changes in lighting conditions and signal reflectively in a given workspace, respectively. Meanwhile, the magnetic field inside buildings is often overlooked as a modality of indoor navigation. The result is that readings from magnetometers, which are present on most modern inertial measurements units (IMUs), are simply ignored in lieu of sensing modalities that are closer to how we perceive the world.

This paper presents a pipeline to generate and validate 3D magnetic field maps, interpret the spatial variation of such maps via a new visual representation, and extract the value of magnetic field maps by leveraging them for three-degree-of-freedom (3DOF) attitude estimation. The maps utilize Gaussian process regression (GPR) to represent the magnetic field based on observations gathered during training flights. The GPR-based maps can then interpolate to provide estimates of the magnetic field at locations that were not directly sampled in the training set. These interpolated estimates of the field serve as a source of magnetic field reference vectors to estimate the attitude of a flight vehicle.

An important aim of this work was to provide a means of leveraging magnetic fields that already exist in every building and improve the indoor navigation of robots. There are two opposing methods of using indoor magnetic fields in the literature. When the goal is to estimate attitude or heading using an indoor magnetic field, some works aim to remove or mitigate anomalies in the magnetic field [4,5,6] or reduce the impact of the magnetometer’s effect on attitude estimates if disturbances are detected [7,8,9,10,11]. In this paradigm, Earth’s magnetic field is seen as the signal, while any disturbances or anomalies are treated as noisy perturbations to be rejected. This methodology assumes that large perturbations are temporary and can have increased estimation errors if disturbances exist for long durations [4]. However, when performing position localization or full-pose (position and attitude) estimation [12,13,14,15,16,17,18,19,20], these “disturbances” become a signal whose spatial variation is needed to distinguish one location from another.

**Figure 1:**
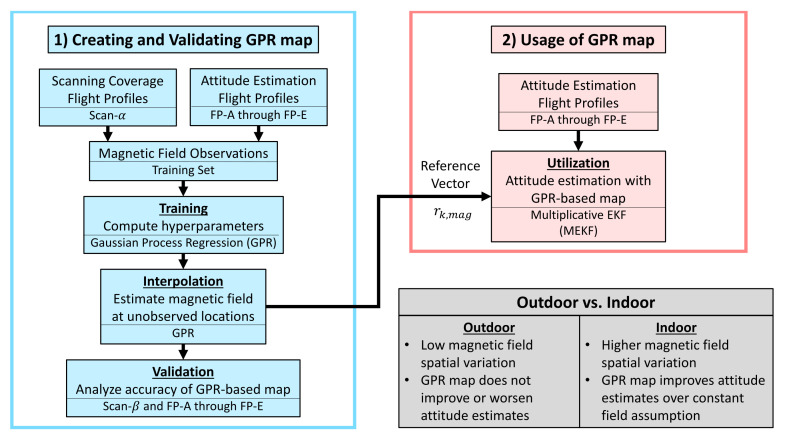
Outline of the main methods of this paper. Several flight profile (FP) trajectories are used to train, validate, and utilize a Gaussian process regression (GPR)-based map.

This paper performs attitude estimation by mapping and leveraging magnetic field disturbances as a complement to works that use magnetic fields for position localization. Other works have shown that using magnetometers for attitude estimation indoors requires more care than outdoor spaces. Here, we investigated if creating magnetic field maps can solve this problem. To test this, we conducted a set of flight experiments both indoors and outdoors to compare the spatial variation of their respective magnetic fields and show how this spatial variation drives the need for mapping. In this paper, the utility of a magnetic field map was measured by its accuracy in representing the local magnetic field and its ability to enable accurate 3DOF attitude estimates. As such, five distinct flight trajectories were flown to analyze the value of both indoor and outdoor magnetic field maps under different flight conditions that excite the roll, pitch, and yaw of a flight vehicle. The outline of our work is summarized in Figure 1.

The contributions of this work are as follows. First, we propose a new way of visualizing 3D magnetic fields that utilizes the interpolative power of GPR-based maps to show the planar direction and downward magnitude of the magnetic field at various altitudes. Next, we present different flight patterns and demonstrate how they can be used to train, validate, and utilize 3D magnetic field maps. Although this work uses GPRs as the interpolative tool of the magnetic field maps, other interpolation methods might still find value in these flight patterns and our training, validation, and utilization techniques. Finally, to our knowledge, no other work has analyzed the accuracy of 3DOF attitude estimation using a single gyroscope, accelerometer, and magnetometer in an environment with the type of spatial variation in the magnetic field in which we tested. With an accurate magnetic field map, one can attain accurate 3DOF attitude estimates in locations with the spatial variation needed to perform magnetic field position localization. We understand that this last contribution comes with many qualifiers, and we will further discuss similar works in the literature and how our work is distinct from theirs in the Related Works Section (Section 2).

The remainder of this paper is structured as follows. Section 2 of this paper outlines related work before mathematical preliminaries are introduced in Section 3. The experimental setup and testing procedure are explained in Section 4. Finally, Section 5 presents results and discussion followed by a conclusion in Section 6.

## 2. Related Work

Previous publications have shown that modalities such as vision [1,2] and wireless technologies [3] can be used for indoor navigation with demonstrations of these methods on UAVs [21,22,23]. However, these modalities add equipment to the test vehicle and sometimes require changes to the workspace (e.g., fiducial markers, ultra-wideband transmitters, etc.) for them to work effectively. Meanwhile, most modern IMUs have magnetometers that can leverage the magnetic fields already present in every building on Earth. Learning to extract value from indoor magnetic fields can improve the indoor navigation capabilities without additional equipment on or off the test vehicle.

Using magnetic fields as a modality is not without its own problems. Previous works have shown that the movement of doors, elevators [19], tool chests [24], and even whiteboards [11] can distort the magnetic field. However, these distortions have been shown to be very localized, while the remainder of the indoor magnetic field has been shown to vary slowly in time [18,19]. Additionally, creating magnetic field maps of large volumes can be computationally expensive [16] if the user is not careful in modeling the magnetic field [24]. Despite this, several previous publications have leveraged the magnetic field of indoor spaces to improve position and attitude estimates for mobile robots such as [17,19] with one dimension of translation, [13,14,15,18,20,25] in planar motion and [12,16] for three dimensions of translation. These works showed that with a magnetic field map of a workspace, it is possible to localize the position of a robot within the given magnetic field map. For localization to work, however, there must be sufficient spatial variation in the magnetic field for any algorithm to distinguish any one location from another.

To our knowledge, there is not yet a consistent method of defining spatial variation in the magnetic field. Reference [4] quantified the change in the magnetic field norm; Reference [18] discussed changes in each component of the magnetic field; Reference [25] used the mean and standard deviation of the change in the magnetic field norm as position changes. By contrast, Vallivaara et al. discussed spatial variation in a binary sense, by assessing if there was sufficient variation to localize or perform SLAM, which can depend on the parameters in the estimation algorithm [13,14]. References [26,27] were works on outdoor magnetic field navigation for fishing vessels and red-spotted newts, respectively. These works described the spatial variation as a gradient of change in the magnetic field strength or direction over some distance. Our work presents a method to visualize the spatial variance of the magnetic field in a workspace and qualitatively compared the spatial variance in different areas.

Unlike position localization, attempts to estimate attitude indoors tend towards detecting and rejecting perturbations in Earth’s magnetic field [4,5,6]. However, the methods from these works do not easily extend to full 3DOF roll, pitch, and yaw estimation and require multiple magnetometers. References [7,8,9,10,11] and others mitigated the effect of disturbances by adjusting the credibility of the magnetometer’s or accelerometer’s measurements when distortions in either were detected. Such works proposed different methods of detecting disturbances, but generally assumed that the disturbance was brief and the magnetic field would return back to some expected value. Reference [28] relaxed this assumption by requiring only that the norm of the magnetic field remain constant over small time periods. However, Reference [28] did not provide 3DOF attitude estimation metrics of their indoor tests to understand how well this method works for indoor navigation. Reference [11] compared their disturbance rejection method to that of [28] and other works with brief, yet large, magnetic field disturbances.

In our work, we performed 3DOF attitude estimation using a gyro, accelerometer, and a single magnetometer despite magnetic field disturbances from buildings and human-made structures. Using magnetic field maps, we leveraged the spatial variation of the field to assist in 3DOF attitude estimation rather than rejecting these features as disturbances. Such capabilities are important for full pose estimation using magnetometers indoors since spatial variation in the magnetic field is desirable for position localization. Although we drew a distinction in our method from the disturbance rejection paradigm, our mapping-based methods can still be paired with the disturbance rejection methods if a magnetic field map is locally inconsistent in a region near doors, elevators, or other mobile ferromagnetic objects. Time-varying magnetic field mapping was addressed in [24], but not in this paper.

Previous works that generated magnetic field maps can be split into two groups. Some performed simultaneous localization and mapping (SLAM) and were able to construct a magnetic field map as they estimated the pose of their vehicle [12,13,14]. Others first had their robot traverse the workspace to learn the magnetic field and construct a map, then utilized this map in a separate trial to perform state estimation or localization [15,16,17,18,19,20,25]. Our work adopted the philosophy of the latter group by performing a set of *training* test flights to construct a magnetic field map followed by a separate set of *utilization* flights meant to assess the accuracy of our attitude estimates by leveraging an existing map. We analyzed the accuracy of our maps by using *validation* test flights and compared two methods of training a GPR-based magnetic field map.

There are different methods of representing magnetic field maps with varying complexities depending on the degrees of freedom of the state to estimate. A magnetic field map with an input of dimension *p* and output of dimension *m* is denoted as a p→m map. Generally, *p* depends on how the vehicle can traverse in space (e.g., p=2 for ground-based, wheeled robots and p=3 for flying vehicles or pedestrian localization), while *m* depends on how many components of the magnetic field are to be tracked. Typically, m=1 when the map gives the norm of the magnetic field for each position, and m=3 when all three components of the magnetic field are tracked. The methods of representing the magnetic field tend to differ based on the size of *p*. Note that the works that tried to detect and mitigate perturbations in Earth’s magnetic field did not create local magnetic field maps [4,5,6,7,8,9,10].

Reference [17] constructed a 1→1 map of the magnetic field’s declination using least squares to interpolate and create unique signatures at select locations of interest. Reference [19] constructed a 1→1 map of the magnetic field norm via linear interpolation. Reference [19] preceded [13,14] in using Gaussian processes to create 2→3 maps. References [20,29] constructed 2→3 and 2→2 maps respectively using bilinear interpolation of the four nearest observations gathered at evenly spaced locations. Reference [18] compared the position localization accuracy of a 2→2 map against a 2→3 magnetic field map by sampling the magnetic field at discrete locations. However, Reference [18] did not interpolate the magnetic field between observed locations, which made their “map” differ from other maps cited in this discussion. In [15,16], Akai and Okazi constructed 2→3 and 3→1 maps, respectively, inside a building to demonstrate the use of Gaussian processes maps in large volumes. Finally, Reference [12] created a 3→3 map also using Gaussian processes.

Linear interpolation is intuitive when *p* = 1 [19], but the bilinear interpolation for *p* = 2 used in [20,29] requires magnetic field observations to be gathered at points on an evenly spaced grid throughout the workspace. Such a constraint is restrictive in how magnetic field map observations are gathered (Section 5.4), is time-consuming for *p* = 3 [16], and does not allow for extrapolation of points outside of those found in the training set (Section 5.1). It is not clear how the *p* = 1 least-squares-based feature selection in [17] could extend to *p* = 2 or *p* = 3. By contrast, Gaussian processes are a prominent way of representing local magnetic fields for p=2 or p=3 as per [12,13,14,15,16]. Many GPR-based applications appear to build on the methods of [30]. Gaussian process regression has no hard constraints on the spatial layout of observations, is able to extrapolate and estimate at locations outside of those found in the training set, as well as provides uncertainty metrics on the confidence of a provided magnetic field estimate. However, GPR comes with a high computational cost as a function of the training set size [16,24]. Due to these listed advantages and their use in other magnetic field mapping works, our work used three independent Gaussian processes to represent a 3→3 magnetic field map.

Kalman filters are a common way to conduct state estimation of dynamic systems: particularly when the rate of change of certain states can be measured or modeled well. Since Kalman filters assume the state and sensor measurements are all Gaussian, particle filters can outperform Kalman filters if parts of the system are non-Gaussian. However, particle filters come at an increased computational cost to track multiple hypotheses of the state, while Kalman filters only work with a single hypothesis. Methods such as TRIAD and derivatives of Wahba’s problem are for attitude *determination* and do not incorporate system dynamics to integrate current estimates forward in time. In this work, we used a multiplicative extended Kalman filter (MEKF), which uses quaternions to represent attitude, is more careful to respect the constraints of rotations than a typical extended Kalman filter, and has been used on many platforms with an IMU [10,31,32,33,34,35]. Here, we used the MEKF derived from [36,37] to perform attitude estimation with a gyroscope, accelerometer, and magnetometer.

## 3. Mathematical Formulation

This section introduces the mathematical preliminaries used throughout the paper. First, we summarize the multiplicative extended Kalman filter (MEKF) from [36,37] that we used to perform attitude estimation from the vehicle’s sensors. Next, we show how the Gaussian processes regression (GPR) method from [30] can be used to generate maps of the magnetic field in an area of interest. Finally, the magnetometer calibration method from [38] is introduced with a nine-parameter sensor model.

### 3.1. Multiplicative Extended Kalman Filter for 3DOF Attitude Estimation

The MEKF is a variation on the extended Kalman filter (EKF) that computes attitude error as the quaternion product of the true attitude and the estimated attitude [36,37]. As with the EKF, the MEKF propagates the current attitude forward in time with a prediction step and uses measurements to correct this prediction in the update step. Estimates of the gyroscope (gyro) bias are used to correct for gyro drift. The formulation of the MEKF presented here was from Chapter 7.1 of [36]. A quaternion was used to represent vehicle global attitude. Instead of updating the four-element quaternion at each step, the MEKF tracks a three-element parameterization of the attitude error used to adjust the global attitude quaternion. As recommended by Section 6.2 of [37], gyro measurements were used for the prediction step instead of a dynamics model of the vehicle. An accelerometer and a magnetometer were used for the correction step.

The MEKF estimates vehicle attitude relative to a world-fixed frame with a quaternion ${\hat{q}}_{k}$ and a gyroscope bias parameter ${\hat{\beta }}_{k}$ at discrete time step *k*. During the prediction step, the gyro measurement was used to integrate the quaternion estimate to the next discrete time step ${\hat{q}}_{k+1}^{-}=\Omega \left({\hat{\omega }}_{k},\Delta t\right){\hat{q}}_{k}^{+}$ where superscript ${}^{-}$ indicates a value immediately after the prediction step and ${}^{+}$ is the value immediately after the update step. During the update step, the MEKF uses a six-component state vector $\Delta {\hat{x}}_{k}={\left[\begin{array}{cc} \delta {\hat{\alpha }}_{k}^{⊤} & \Delta {\hat{\beta }}_{k}^{⊤} \end{array}\right]}^{⊤}$, where $\delta {\hat{\alpha }}_{k}$ is the three-component attitude estimate error vector and $\Delta {\hat{\beta }}_{k}=\beta_{k}-{\hat{\beta }}_{K}$ is a three-component gyro bias estimate error vector, to correct the prediction. In effect, the update step computes ${\hat{q}}_{k+1}^{+}={\hat{q}}_{k+1}^{-}+\frac{1}{2}\Xi \left({\hat{q}}_{k+1}^{-}\right)\delta {\hat{\alpha }}_{k}$ for the attitude and ${\hat{\beta }}_{k+1}^{+}={\hat{\beta }}_{k}^{-}+\Delta {\hat{\beta }}_{k}^{-}$ for the gyro bias.

As with all Kalman filters, the MEKF also keeps track of its estimate uncertainty with a 6×6 covariance matrix $P_{k}$ where the six-dimensional estimate is assumed to be drawn from a Gaussian distribution $\Delta {\hat{x}}_{k}∼N\left(\Delta {\hat{\bar{x}}}_{k},P_{k}\right)$.

#### 3.1.1. MEKF Prediction Step

The MEKF prediction step uses gyro data to march the current estimate forward. Gyroscope values are modeled as:

(1) $$\begin{array}{c} {\tilde{\omega }}_{k}=\omega_{k}+\nu \;\;\nu ∼N\left(0,Q_{k}\right) \end{array}$$

where $\omega_{k}$ is the true angular velocity and $Q_{k}$ is the process noise matrix. The MEKF first offsets the measured gyro value by current gyro bias estimate ${\hat{\omega }}_{k}={\tilde{\omega }}_{k}-{\hat{\beta }}_{k}$ where ${\tilde{\omega }}_{k}$ is the raw gyro measurement and ${\hat{\omega }}_{k}$ was offset by our bias estimate ${\hat{\beta }}_{k}$. We then updated the quaternion and gyro bias with:

(2) $$\begin{array}{cc} {\hat{q}}_{k+1}^{-} & =\Omega \left({\hat{\omega }}_{k},\Delta t\right){\hat{q}}_{k}^{+}\\ {\hat{\beta }}_{k+1}^{-} & ={\hat{\beta }}_{k}^{+} \end{array}$$

where $\Omega ({\hat{\omega }}_{k},\Delta t)$ was defined in Chapter 7.1.2 of [36] and depends only on the bias-adjusted gyro measurement ${\hat{\omega }}_{k}$ and the amount of time given by discrete time step Δt. Note the gyro bias estimate is unchanged during the prediction step.

The covariance matrix becomes:

(3) $$P_{k+1}^{-}=\Phi_{k}P_{k}^{+}\Phi_{k}^{⊤}+YQ_{k}Y^{⊤}$$

where $\Phi_{k}=\Phi_{k}\left({\hat{\omega }}_{k},\Delta t\right)$ is a state transition matrix that depends on ${\hat{\omega }}_{k}$ and Δt, while Y is a constant matrix. $Q_{k}=Q_{k}\left(\sigma_{u},\sigma_{v},\Delta t\right)$ is a process noise matrix that depends on gyroscope noise parameters $\sigma_{u}$ and $\sigma_{v}$ and the change in time Δt. All three of these matrices were defined in Chapter 7.1.2 of [36].

#### 3.1.2. MEKF Update Step

The update step receives accelerometer ${\tilde{z}}_{k,accl}$ and magnetometer ${\tilde{z}}_{k,mag}$ measurements, compares these to their respective “reference vectors” in world frame $r_{k,accl}$ and $r_{k,mag}$, and uses the difference between the measurement and reference vector to update the estimate. Reference vectors are effectively what we expected each sensor to measure given the current vehicle state estimate. It was assumed that the gyro data used in the prediction step be measured at the same time as the accelerometer and magnetometer data. On our test UAV, the magnetometer was sampled less frequently than the accelerometer and gyroscope. As such, we used a sequential update method explained in Chapter 7.1.3 of [36] so that the matrices used in the update step need not change size depending on the availability of magnetometer data.

First, we define a rotation matrix $A(q_{k})$ that transforms a vector represented in the world frame to the same vector represented in the vehicle body frame. We then define the Kalman gain:

(4) $$K_{k}=P_{k}^{-}H_{k}^{⊤}{\left[H_{k}P_{k}^{-}H_{k}^{⊤}+\sigma_{\_}I_{3\times 3}\right]}^{-1}$$

where $I_{3\times 3}$ is the 3×3 identity matrix, and the 3×6 sensitivity matrix $H_{k}$ is:

(5) $$H_{k}=\left[{\left[A\left({\hat{q}}_{k}^{-}\right)r_{k,\_}\right]}^{\times }\;0_{3\times 3}\right]=\frac{\partial h_{\_}}{\partial (\Delta x)}=\left[H_{\alpha }\;H_{\beta }\right].$$

$\sigma_{\_}$ is a measurement noise parameter that depends on which sensor is being used (i.e., $\sigma_{accl}$ or $\sigma_{mag}$) and $r_{k,\_}$ is the corresponding accelerometer or magnetometer reference vector. $H_{k}$ is a Jacobian matrix of the measurement model:

(6) $$\begin{array}{c} {\tilde{z}}_{k,mag}=h_{mag}\left(q_{k}\right)+\eta_{mag}\\ {\tilde{z}}_{k,accl}=h_{accl}\left(q_{k}\right)+\eta_{accl} \end{array}$$

where

(7) $$\begin{array}{c} \eta_{mag}∼N\left(0,\;\sigma_{mag}I_{3\times 3}\right)\\ \eta_{accl}∼N\left(0,\;\sigma_{accl}I_{3\times 3}\right). \end{array}$$

In general, $h_{mag}$ varies as a function of position if the magnetic field is not constant. However, this has no effect on the Jacobian $H_{k}=\frac{\partial h_{\_}}{\partial (\Delta x)}$ since position is not part of the state Δx. The sensor-based subscript (i.e., ${}_{mag}$ or ${}_{accl}$) was omitted from $H_{k}$ for brevity. Finally, the operator ${\left[a\right]}^{\times }$ takes a vector $a={\left[a_{x}\;a_{y}\;a_{z}\right]}^{⊤}$ as the input and returns a skew-symmetric cross-product matrix where:

(8) $${\left[a\right]}^{\times }=\left[\begin{array}{ccc} 0 & -a_{z} & a_{y}\\ a_{z} & 0 & -a_{x}\\ -a_{y} & a_{x} & 0 \end{array}\right]\;S.T.\;a\times b={\left[A\right]}^{\times }b.$$

The estimate error state is then updated with:

(9) $$\begin{array}{cc} \epsilon_{k,\_} & ={\tilde{z}}_{k,\_}-A\left({\hat{q}}_{k}^{-}\right)r_{k,\_}\\ \Delta {\hat{x}}_{k}^{+} & =\Delta {\hat{x}}_{k}^{-}+K_{k}\left[\epsilon_{k,\_}-H_{k}\Delta {\hat{x}}_{k}^{-}\right] \end{array}$$

where $\epsilon_{k,\_}$ is often denoted the “residual” or “innovation” and ${\tilde{z}}_{k,\_}$ is either the accelerometer or magnetometer measurement. $\Delta {\hat{x}}_{k}^{+}$ is used to update the estimate quaternion ${\hat{q}}_{k}^{+}$ and gyro bias estimate ${\hat{\beta }}_{k}^{+}$ as specified in Chapter 7.1.1 of [36]. Finally, the covariance matrix is updated with:

(10) $$P_{k}^{+}=\left[I_{6\times 6}-K_{k}H_{k}\right]P_{k}^{-}.$$

### 3.2. Gaussian Process Regression

The purpose of the GPR-based map is to take observations around the flight space and interpolate these measurements to predict the magnetic field at any point in and around the workspace. When estimating attitude, the GPR-based map is queried at the vehicle’s location to provide a magnetic field reference vector $r_{k,mag}$ to the MEKF.

Gaussian process regression uses a “training set” of observations to make predictions of the magnetic field. The predictions tend to only be accurate near positions used in the training set. Each observation has a 3D position and the three components of the magnetic field observed at position (${\tilde{B}}_{x}$, ${\tilde{B}}_{y}$, ${\tilde{B}}_{z}$). For this work, we used three independent Gaussian processes, each with a 3D input (the position) and a scalar prediction as the output (the *x*, *y*, or *z* component of the magnetic field). As such, for a training set with *n* observations, we defined the design matrix $x\in R^{3\times n}$ as a collection of the *n* 3D positions and defined vectors ${\tilde{y}}_{x}$, ${\tilde{y}}_{y}$, ${\tilde{y}}_{z}\in R^{n}$ as the corresponding magnetic field observations for ${\tilde{B}}_{x}$, ${\tilde{B}}_{y}$, and ${\tilde{B}}_{z}$, respectively.

In [30], Rasmussen and Williams defined a Gaussian process as a distribution over functions written as:

(11) $$f\left(x\right)\;∼\;\mathrm{GP}\left(0,\;k(x,x^{\prime })\right)$$

where 0 is the zero mean function, *k* is a covariance function (or kernel), and x, $x^{\prime }\in R^{3}$ are the 3D positions. The covariance function quantifies correlation, or similarity, in observations based on their spatial proximity. For this work, we used three separate Gaussian processes ${\mathrm{GP}}_{x}$, ${\mathrm{GP}}_{y}$, and ${\mathrm{GP}}_{z}$ that have a zero mean function and a squared exponential covariance function. Given a training dataset and a set of hyperparameters, each Gaussian process can be queried at a 3D location to provide a prediction of a single component of the magnetic field along with the uncertainty of each prediction. The “map” of the magnetic field is actually these three Gaussian processes with their corresponding training sets $D_{x}=\left(X,{\tilde{y}}_{x}\right)$, $D_{y}=\left(X,{\tilde{y}}_{y}\right)$, and $D_{z}=\left(X,{\tilde{y}}_{z}\right)$ and their respective optimized hyperparameters.

The hyperparameters are adjustable terms from the covariance function. With a squared exponential covariance function, the hyperparameters are a length scale and a signal standard deviation. Each of the three Gaussian processes had distinct hyperparameters computed by minimizing the negative log marginal likelihood over their corresponding training datasets. Hyperparameter optimization was performed using the *gpml-matlab* (*gpml-matlab* was created by Carl Edward Rasmussen and Hannes Nickisch toolbox (http://www.gaussianprocess.org/gpml/code/matlab/doc/, accessed on 19 September 2021).

In this paper, we refer to the three Gaussian processes as a single object (e.g., “map”, “magnetic field map”, “GPR-based map”, “GPR”) since they were used together to predict the full magnetic field vector at a desired location. These predictions are used by the MEKF as magnetic field reference vectors $r_{k,mag}$ and to assess the accuracy of the GPR-based maps via a validation set per Section 5.1 and Section 5.4. Finally, the predictions are used to visualize the magnetic field of our workspaces in Section 5.2.

### 3.3. Magnetometer Measurement Model

To calibrate the magnetometer on our flight vehicle, we used the model and procedure from [38]. Measurements from a three-axis magnetometer can differ from the actual magnetic field because of noise, sensor bias, nonunit scaling factors, and nonorthogonalities in the alignment of the magnetometer’s measurement axes. These imperfections are captured by the following model:

(12) $$\begin{array}{c} {\tilde{B}}_{x}=aB_{x}+x_{0}+\eta_{x} \end{array}$$

(13) $$\begin{array}{c} {\tilde{B}}_{y}=b\left({\tilde{B}}_{y}\cos\left(\rho \right)+B_{x}\sin\left(\rho \right)\right)+y_{0}+\eta_{y} \end{array}$$

(14) $$\begin{array}{c} \begin{array}{cc} {\tilde{B}}_{z} & =c(B_{x}\sin\left(\lambda \right)+B_{y}\sin\left(\phi \right)\cos\left(\lambda \right)\\ & +B_{z}\cos\left(\phi \right)\cos\left(\lambda \right))+z_{0}+\eta_{z} \end{array} \end{array}$$

where (${\tilde{B}}_{x}$, ${\tilde{B}}_{y}$, ${\tilde{B}}_{z}$) are measured values, ($B_{x}$, $B_{y}$, $B_{z}$) are the true magnetic field, (*a*, *b*, *c*) are scaling factors, ($x_{0}$, $y_{0}$, $z_{0}$) are constant bias, ($\eta_{x}$, $\eta_{y}$, $\eta_{z}$) are zero-mean noise, and (ρ, λ, ϕ) are angles quantifying the nonorthogonality of the axes (Figure 1 in [38]). The goal is to estimate $\alpha ={\left[a,b,c,x_{0},y_{0},z_{0},\rho ,\lambda ,\phi \right]}^{⊤}$ by minimizing:

(15) $$\begin{array}{cc} \Delta B & =B_{R}^{2}-B^{2}\\ \; & =B_{R}^{2}-\left(B_{x}^{2}+B_{y}^{2}+B_{z}^{2}\right)\\ \; & =B_{R}^{2}-g\left({\tilde{B}}_{x},{\tilde{B}}_{y},{\tilde{B}}_{z},\alpha \right) \end{array}$$

where $B_{R}$ is the magnitude of the magnetic field reference vector obtained from the GPR-based map and g() is a nonlinear function obtained by re-arranging Equations (12) through (14) for ($B_{x}$, $B_{y}$, $B_{z}$). The estimated α is obtained by iterative nonlinear least squares with $\alpha_{0}={\left[1,1,1,0,0,0,0,0,0\right]}^{⊤}$ as an initial condition.

## 4. Experimental Setup and Procedure

The purpose of our experiments was to gather sensor data to create magnetic field maps, assess their accuracy in representing the local magnetic field, and analyze their ability to enable the 3DOF attitude estimation of a UAV. To this end, we created coverage flight patterns (Scan-α and Scan-β) to study different ways of gathering observations to train magnetic field maps and investigate the interpolation and extrapolation capabilities of GPR-based maps. Then, we flew five different flight profiles (FP-A through FP-E) that distinctly excited the roll, pitch, and yaw of the flight vehicle to provide different test cases for attitude estimation. These profiles were flown both indoors and outdoors to investigate how spatial variation in the magnetic field affects the utility of our magnetic field maps.

### 4.1. Equipment, Facilities, and Setup

**Figure 2:**
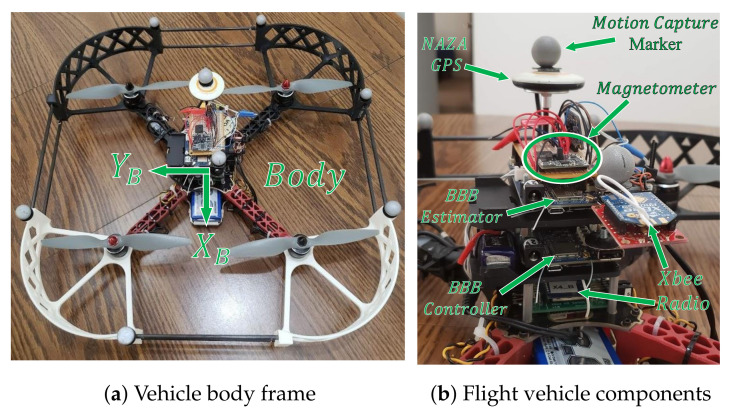
Flight vehicle.

Our experimental flight vehicle, shown in Figure 2, is a multirotor equipped with a gyroscope, accelerometer, and magnetometer. The multirotor used here were introduced in Section IV of [39] and flies an MPU9250 IMU (gyro, accelerometer, magnetometer) and BMP280 barometer. Since the work in [39], a second BeagleBone Blue microprocessor, a PNI RM3100 magnetometer, and a DJI Naza-M V2 GPS unit were added to the vehicle. In addition, the flight controller was changed to the Robotics Cape autopilot (*RC Pilot*) (*RC Pilot* was created by StrawsonDesign (https://github.com/StrawsonDesign/rc_pilot, accessed on 19 September 2021)) open-source flight controller.

**Figure 3:**
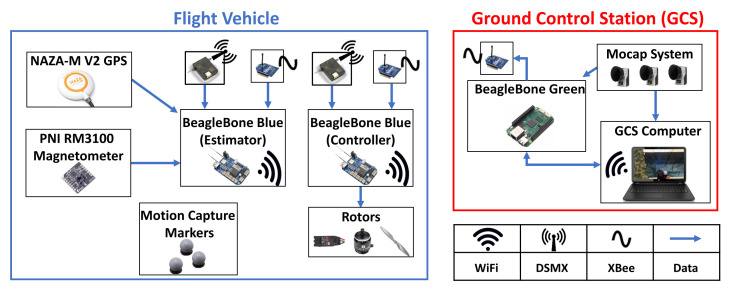
Diagram of the devices used to the gather experimental data.

Figure 3 shows the major components of the experimental setup. The flight vehicle was equipped with two BeagleBone Blue (BB-Blue) microprocessors both running RC pilot. The controller BB-Blue uses pregenerated trajectories as guidance and uses a linear quadratic regulator (LQR) controller to maneuver the vehicle along the desired trajectory. The controller used in this paper was a modified version of the LQR controller from [40]. For navigation, the MPU9250 IMU has a built-in processor that provides roll and pitch estimates, while motion capture position and yaw estimates are streamed from the ground station to the vehicle via wireless XBee radios during each flight.

The estimator BB-Blue logs data from the NAZA-M V2 GPS, PNI RM3100 magnetometer, MPU9250 IMU (gyro and accelerometer), BMP280 barometer, and Qualisys motion capture with a timestamp from when each respective value was logged. The MPU9250 IMU and BMP280 barometer are included with each BeagleBone Blue and are thus not included individually in Figure 3. The IMU was sampled at 200 Hz, the magnetometer at approximately 144 Hz, the barometer at 20 Hz, and the GPS at 4 Hz. The Qualisys (outdoor) motion capture pose estimates were computed at 100 Hz, while the Optitrack (indoor) system provides ground truth at 120 Hz. The GPS and barometer data were not used in this work.

The LQR controller from [40] did not use integrator terms on position, altitude, or yaw angle. The controller was not tuned rigorously for this work, so there tended to be a slight, but slow decrease in altitude during each flight. In addition, the guidance method assumed the processor updates the outputs at 200 Hz consistently, which is not true in reality. The result is that the desired position, velocity, and attitude fed to the controller is sometimes behind a location the drone has already passed. This causes some undesirable motion from a control perspective, but actually presents interesting oscillatory features for our MEKF to track. For each flight, the vehicle was placed near the motion capture origin with the +X axis of the vehicle’s body frame roughly aligned with the +X axis of the motion capture origin. To prevent large, unpredictable motion during takeoff, the controller’s initial reference XY position and heading were the initial position and heading of the vehicle before takeoff

The Network Time Protocol (NTP) was used to synchronize all data-gathering processors to ensure proper temporal correspondence between sensor data gathered onboard the vehicle and the ground truth from the motion capture system. There was a ∼40 ms delay when wirelessly transmitting the motion capture pose from the ground station to the flight vehicle via Xbee radio. In order to correlate onboard sensor data to motion capture ground truth values, all BeagleBone processors (the two BB-Blues on the vehicle and the BB-Green on the ground station per Figure 3) used NTP to synchronize their local clock with the ground station’s clock during the initial setup. Each BeagleBone then recorded their NTP clock offset from the ground station clock at the start and end of each flight trial. These offsets were linearly interpolated for all other points throughout the flight to serve as a method of synchronization between sensor data captured onboard the vehicle and motion capture data captured on the ground station *before* it was sent over the wireless XBee radio channel and subject to the 40 ms delay.

For the outdoor and indoor flight test sessions, a single gyro and accelerometer calibration was performed during the initial setup to remove any large offsets in either sensor. Calibration bias and scaling factors were automatically applied to all raw values for both sensors as a feature of the MPU9250 IMU. The outdoor dataset used in this paper was not originally meant for an analysis of magnetic fields. As such, no magnetometer calibration procedure was performed before the outdoor flight data were gathered. Since the indoor data were gathered specifically for this paper, we used the calibration method from [38]. Table 1 shows the scaling factor, bias, and nonorthogonality metrics for the PNI RM3100 applied to the magnetometer measurements for the indoor flight tests. A perfect sensor would have scale factors of 1 and bias and nonorthogonalities of 0. Even though we did not have a calibrated magnetometer for the outdoor readings, Table 1 gives us confidence that the RM3100 provides good measurements with uncalibrated errors that are small relative to the 50 μT field at the outdoor flight space.

All outdoor tests were conducted at the University of Michigan’s outdoor netted drone facility, M-Air, equipped with 30 Qualisys motion capture cameras for ground truth position and attitude. The indoor flight tests were conducted in the Robot Fly Lab in the Ford Motor Company Robotics Building equipped with 8 Optitrack motion capture cameras for the ground truth. The outdoor data were collected in a single August 2020 evening in M-Air. The indoor data were gathered in March 2021 inside the Robot Fly Lab. The usable volume for the indoor flight lab space is smaller than that of M-Air. As such, the flight profiles used outdoors (Section 4.2) covered larger areas than their indoor equivalents (Section 4.3)

For the outdoor flights, the motion capture origin was set at the center of M-Air, which was surveyed to be 42${}^{\circ }$17’39.95257” N, 83${}^{\circ }$42’37.59818” W. According to Google Earth, the center of M-Air has an altitude of 270m above sea level. M-Air has pillars that align with geographic north–south and east–west. Using these pillars, the +X axis of the motion capture system was aligned with geographic north (by visual inspection) and the +Z motion capture axis aligned with the gravity vector using a leveling tool.

For indoor flights, the +X axis of the motion capture origin was aligned with the long axis of the indoor flight space. There is no special relationship between the indoor fly lab’s selected origin and Geographic North. To achieve consistency between flights, we marked the position and orientation of the desired motion capture origin on the ground. The +X axis of the *indoor* motion capture origin was aligned with this tape marker via visual inspection while the +Z axis of the motion capture system was aligned with the gravity vector using a leveling tool.

Motion capture position and attitude estimates are given with respect to a virtual rigid body defined from a set of motion capture markers on the vehicle. The body frame for the virtual vehicle is the centroid of all motion capture markers used to define the rigid body and does not necessarily coincide with the center of mass of the actual vehicle. For outdoor data, the orientation of the virtual rigid body was aligned with the physical vehicle through select placement of motion capture markers, a feature of the Qualisys motion capture system. The indoor Optitrack motion capture system has no feature to align the virtual rigid body with the physical vehicle, but our motion capture targets were only placed once for all indoor flights, so any systematic bias was consistent in all data gathered. To characterize the performance of our attitude estimator under different flight maneuvers, each flight test followed a trajectory from one of the flight profiles detailed below.

### 4.2. Outdoor Flight Profiles

**Figure 4:**
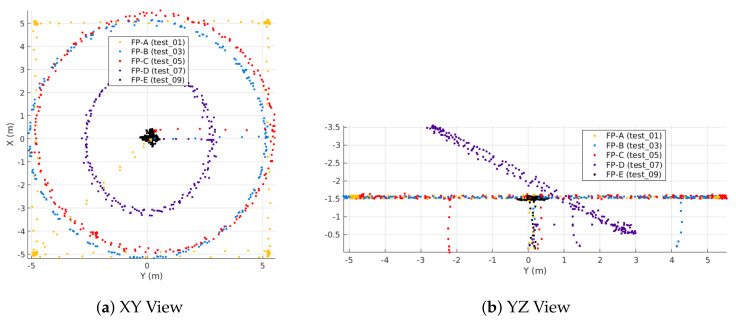
Five outdoor flight profiles.

To investigate the value of magnetic field maps for indoor attitude estimation, the multirotor flew five different flight profiles, each meant to excite roll, pitch, and yaw in different ways. Flight Profile A (FP-A) is a square trajectory where the vehicle hovers at each corner of the square. This flight profile offers large translations with constant altitude and heading. Flight Profiles B and C are level circle trajectories with large, continuous translations, constant altitude, and a desired heading that is always tangent to the circle. FP-B has a slower tangential speed than FP-C. Flight Profile D is an inclined circle similar to B and C, but altitude is no longer constant. Finally, profile E is an angular oscillation test featuring small translation, but large oscillations in roll and pitch. All trajectories were flown at an altitude of 1.5 m except FP-D (the inclined circle) with the desired altitude varied from 0.7 m to 3.3 m. Figure 4 shows the five outdoor flight profiles. All outdoor flight tests have two-digit identification numbers per Table 2.

FP-A flown outdoors has side length 10 m. The vehicle is commanded to hover at each corner of the square for 10 s followed by a 10 s cubic spline translation to the next corner. From the takeoff point, the vehicle moves to the −X,−Y (southwest) corner, then traces a square by moving +X (north), +Y (east), −X (south), then −Y (west) two times before returning to the origin to land. Cubic spline velocities start and end at rest while position constraints change as appropriate to traverse the square resulting in a maximum commanded speed of 1.5 m/s.

Circular profiles FP-B and FP-C have turning radius 5 m, while FP-D is inclined with respect to the horizontal plane by 30 and has turning radius 3 m. FP-B and FP-C maintain an altitude of 1.5 m, while the inclined circle’s altitude varies from 0.7 m to 3.3 m. The tangential velocity of Profiles B and D are both “slow” at 1 m/s, while Profile C has a commanded tangential velocity of 3 m/s. For all three circular trajectories, the UAV heading is directed to follow a tangent to the circular path.

Flight Profile E follows a sinusoidal desired position in the *X* and *Y* axes. The net result is small linear motion and fast changes in vehicle roll and pitch. The sinusoidal reference trajectory has a 0.3 m amplitude with a frequency of 3 Hz. In each trial of FP-E, the vehicle oscillates along the pitch axis (±X direction) first for 90 s followed by oscillations in the roll axis (±Y direction) for the same duration. The UAV is commanded to maintain a constant heading throughout the flight and remain stationary for 5 s between roll and pitch oscillations to decouple motion in each axis.

Two three-minute trials of each flight profile were conducted with all sensors sampled per Section 4. One trial from each profile was used to create the GPR-based map (training data), while the other was used to perform attitude estimation with an MEKF (utilization data). The specific trials used for training and utilization are shown in Table 2. For the training data, all observations were downsampled to 1 Hz before being fed into the GPR. In the case of the utilization data, the MEKF was given all sensor data at the respective sampling rates.

The flight profiles in this work were meant to capture different types of flight methods. FP-A has short periods of motion with long segments of stationary hover that emulate surveillance-based applications of multirotors. This trajectory enables the drone to experience the planar spatial variation of the workspace by covering the span of the workable area. FP-B was meant to emulate the continuous motion of fixed-winged vehicles, but we settled on a circular trajectory due to the space constraints of our workspace. In retrospect, an elongated and beveled rectangular circuit would allow for periods of straight flight with some banked turns to better represent fixed-winged flight. FP-C is a more aggressive version of FP-B, while FP-D was designed to capture the vertical spatial variation of the workspace. Finally, FP-E is a stress test of roll and pitch estimation, but not a typical maneuver one would expect a flight vehicle to perform.

Our vehicle and its controller are not able to safely execute aggressive maneuvers as seen in drone racing or in acrobatic drones. Though such cases would be good stress tests for the attitude estimation portion, they were not achievable with our current setup. As such, we used FP-C and FP-E as stand-ins for “aggressive” flight maneuvers within the capabilities of our system.

### 4.3. Indoor Flight Profiles

The five flight profiles from the outdoor dataset were repeated inside the Robot Fly Lab with the following differences. To distinguish between outdoor and indoor trajectories, let FP-A denote the outdoor level square, while FP-In-A the indoor variant; analogous notation was adopted for the other four flight profiles. The indoor profiles have smaller X−Y footprints to fit the indoor flight space, so more laps were traversed to achieve the desired three-minute flight duration.

**Figure 5:**
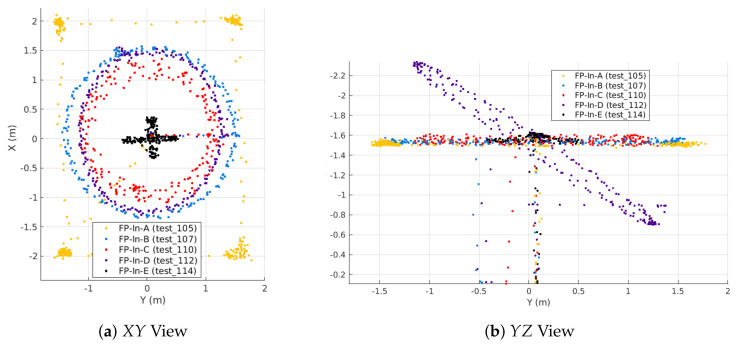
Five indoor flight profiles.

FP-In-A is a rectangle with a 4m side length in the *X* direction and a 3 m side length in *Y*; it is otherwise the same as FP-A. FP-In-B (slow level circle) has a 1.5 m radius, but is otherwise identical to FP-B. FP-In-C (fast level circle) has a 1m radius and a commanded tangential velocity of 1.5 m/s, but is otherwise identical to FP-C. FP-In-D (slow inclined circle) has a 1.5m radius and 30 inclination, which bounds its commanded altitude between 0.85 m and 2.15 m. FP-In-E (stationary oscillations) is identical to the outdoor FP-E. Figure 5 shows the five indoor flight profiles. All flight tests conducted indoors have a three-digit number starting with a “1” per Table 3.

**Figure 6:**
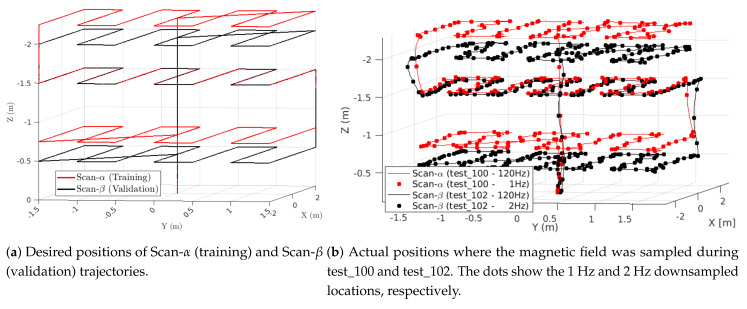
Desired positions of Scan-*α* and Scan-*β*: coverage patterns to train and validate the magnetic field map for the indoor flights.

Two additional trajectories (Scan-α and Scan-β) were flown indoors to train and validate the GPR-based magnetic field map. Both are a lawnmower coverage pattern in the X−Y plane at three different altitudes. Scan-α (training set) was flown at 0.75 m, 1.5 m, and 2.25 m altitudes, while Scan-β (validation set) was flown at 0.5 m, 1.5 m, and 2 m altitudes. The goal was for the validation set to have an altitude that forced the GPR to extrapolate (0.5 m), interpolate (2 m), and match (1.5 m) the training set altitudes. At each altitude, the drone flew a lawnmower pattern in the X−Y plane by traversing from −2 m to +2 m in the *X* direction, then changing the *Y* position from −1.5 m to +1.5 m in 0.5 m increments. Similar coverage patterns have been used in other magnetic field mapping works [41,42,43]. Figure 6 shows the desired positions and actual flight position Scan-α (test_100) and Scan-β (test_102) trajectories. In Figure 6b, the lines show the ground truth position of the vehicle at 120 Hz, while the dots are downsamples of the position used to train (downsampled to 1 Hz) and validate (downsampled to 2 Hz) the GPR-based map.

Two trials of each flight profile were conducted with all sensors being sampled at the aforementioned frequencies (Section 4). For the indoor data, we trained the GPR in two different ways. One methodology is to train the GPR using the Scan-α lawnmower pattern, and the alternative is to train the GPR using one trial each of the five indoor flight profiles (FP-In-A through FP-In-E), as summarized in Table 3. In both cases, we used both the Scan-β trajectory and separate tests of the FP-In flights to validate the map. Below, we use these two methods of training the GPR to analyze which yields more accurate maps (Section 5.1 and Section 5.4). In general, training tests were downsampled to 1 Hz, while validation sets were downsampled to 2 Hz. The one exception is when training with FP-In-A, we downsampled to 2 Hz to provide better coverage along the edges of the planar rectangle. The FP-In-A locations shown in Figure 5 were downsampled to 2 Hz, while all others were at 1 Hz.

### 4.4. Creating the GPR-Based Magnetic Field Reference Map

To create the GPR-based magnetic field map, we first sampled the magnetic field at the desired locations to form a training dataset. For this, we split the flight tests into three sets: GPR *training*, GPR *validation*, and MEKF *utilization*. Table 2 shows which tests were used in each respective set for the outdoor dataset, while Table 3 shows analogous data for the indoor dataset. At the IMU 200Hz sampling rate, each 3 min flight generated over 40,000 datasets. However, many of these points are spatially redundant since the vehicle does not move very far in 1/200 of a second. In order to maintain efficient GPR execution, all training datasets were downsampled to 1 Hz. The exception is FP-In-A, which was downsampled to 2 Hz to provide better coverage on the edges of the planar rectangle. The outdoor GPR map uses 1157 observations from the five outdoor flight profiles. Each point in Figure 4 is an observation used to create the outdoor map. The indoor map was trained in two different ways, either using the ∼250 observations from one of the 2 Scan-α tests (Figure 6b) or using 1298 observations from the indoor flight profiles (Figure 5).

For each map, the observations that made the training set were used to train three separate Gaussian processes. Each observation has a 3D input (x,y, and *z* position of the observation) and a 1D output (a single component of the 3D magnetometer measurement). Once trained, each Gaussian process can be queried to give the X,Y, or *Z* component of the magnetic field at any desired position, including locations that were not directly observed in the training set. In effect, we used the GPR to interpolate and predict the magnetic field at unobserved locations in each flight space. Although three independent Gaussian processes performed regression for each of the X,Y, and *Z* components of magnetic field, we refer to the composition of these as a single unit (e.g., “GPR”, “map”, “GPR-based map”, etc.).

The open-source toolbox *gpml-matlab* (Section 3.2) was used for training and querying the GPR. A squared exponential covariance function was used with a Gaussian likelihood. The hyperparameters (i.e., characteristic length scale, signal and noise standard deviations) for all three Gaussian processes were computed separately by minimizing the log marginal likelihood. The *gpml-matlab* toolbox implements the methods from [30].

### 4.5. Querying the WMM Magnetic Reference Map

The World Magnetic Model (WMM), built by the British Geological Survey group, models Earth’s magnetic field [44]. A similar model called the International Geomagnetic Reference Field (IGRF) gives the same reference field values at M-Air within 0.01 μT.

WMM uses observations of Earth’s magnetic field from Earth-orbiting spacecraft to construct a model of the magnetic field and how it will change over the course of five years. The model is most accurate on the year the new version is released, and accuracy degrades over five years until the next release is available. WMM2020 was released in December of 2020. Although our *outdoor* tests were performed in August 2020, we used WMM2020 rather than its predecessor, WMM2015. For this work, we used WMM to provide a magnetic reference field value $r_{k,mag}$ that was assumed to be constant at all locations throughout the workspace since WMM gives the same magnetic field values for positions within 10m of the outdoor workspace origin. This is why we refer to WMM as our low-resolution source, while the GPR-based map is our high-resolution source of magnetic field reference vectors $r_{k,mag}$.

WMM was not used as a source of reference vectors for the indoor analysis since its predictions were not accurate for the values measured inside. Instead, to emulate the low resolution of WMM, we queried the GPR at a single location indoors and assumed the magnetic field as constant for all points in the workspace.

## 5. Results and Discussion

In this section, we first assess the accuracy of the GPR-based magnetic field map by analyzing its error against a validation set. Since the outdoor dataset did not have a calibrated magnetometer, this accuracy analysis was only performed on the indoor Robotic Fly Lab data. Next, we present a novel method of visualizing 3D magnetic fields in Section 5.2 to highlight the spatial variation differences between the indoor and outdoor magnetic fields. This analysis was used to explain the trends seen in Section 5.3, where we demonstrate the value of magnetic field maps on 3DOF attitude estimation in regions with nonconstant magnetic fields. In Section 5.4, we compare the accuracy of GPR maps trained on the observations from the Scan-α coverage pattern against the maps trained from the indoor flight profiles. Finally, Section 5.5 summarizes how magnetometers can assist with roll and pitch estimates typically reserved for the accelerometer unit to handle.

### 5.1. Accuracy of GPR-Based Magnetic Field Map

This section analyzes the accuracy of the GPR when used to map the magnetic field inside the Robotic Fly Lab. The GPR-based map is capable of accurately estimating the magnetic field even when extrapolating outside of and interpolating between observed regions.

**Figure 7:**
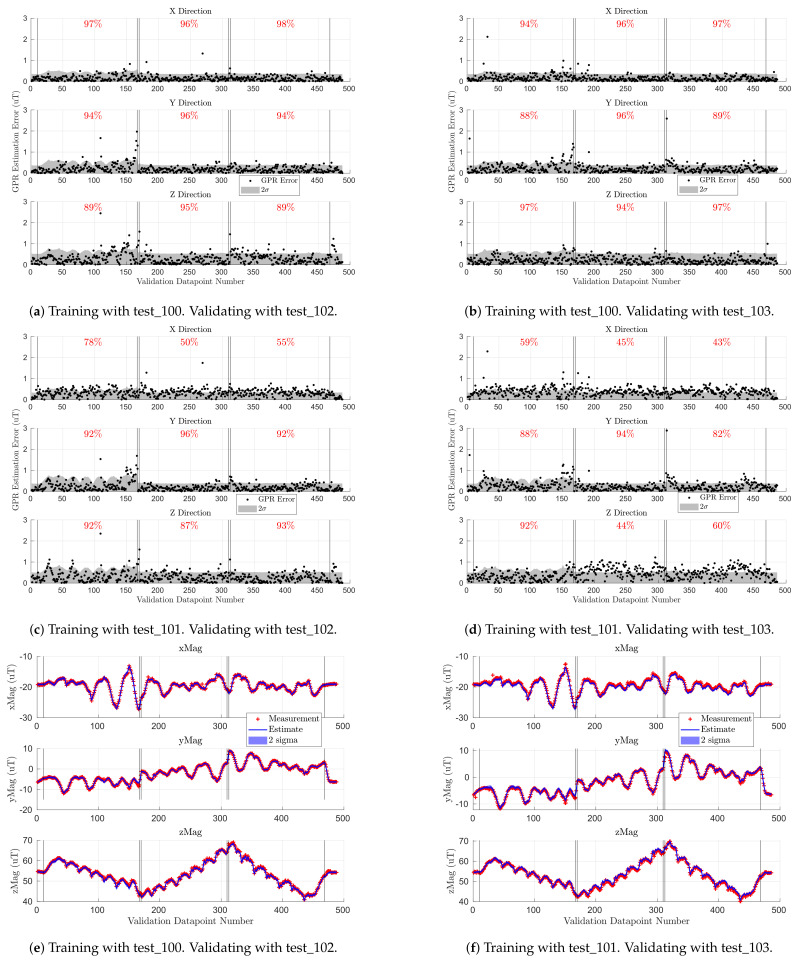
GPR-based magnetic field map error on ∼490 validation points from Scan-*β*. In order, the large segments separated by vertical bars are at altitudes 0.5 m, 1.5 m, and 2 m, respectively. For Figure 7a–d, the red percentage value shows how often the error is within the 2*σ* uncertainty for each altitude segment.

To analyze the accuracy of the magnetic field map, we created a GPR-based map with observations from a Scan-α flight test and checked its accuracy against validation data from a Scan-β test (see Section 4.3). Per Section 4.4, the GPR for the indoor map was created by taking ∼250 observations from the Scan-α flight test_100 or test_101. Figure 7 shows the ${\tilde{B}}_{x}$, ${\tilde{B}}_{y}$, and ${\tilde{B}}_{z}$ estimation error at the ∼490 locations observed in the Scan-β validation flight tests (see Figure 6). The vertical lines in each subplot group the flight into three constant-altitude sections separated by a few observations during altitude changes. Observations at 0.5 m are indexed from 10 to 166, 1.5 m from Indices 169 to 309, and 2.0 m from Indices 312 to 468. The segments from Indices 0 to 10 and 468 onward represent the takeoff and landing sequences, respectively. The black dots represent the GPR estimation error, while the gray fill shows two standard deviations (2σ) of the GPR’s uncertainty at each location. The red percentage in each constant-altitude section quantifies how often the error is within the gray uncertainty region. Figure 7e,f show estimated and measured magnetic field values at each location, which were used to compute the errors shown in Figure 7a,d, respectively. Here, each red cross is a measurement from the validation set, while the blue line and shading are the GPR prediction and uncertainty, respectively. Figure 7e,f are qualitatively similar to the other two training/validation pairs.

The GPR for each axis generally had less than 1 μT of error over all validation measurements. The Scan-α training trajectory sampled points at altitudes 0.75 m, 1.5 m, and 2.25 m, while the Scan-β validation set flew at 0.5 m, 1.5 m, and 2.0 m. As such, the GPR must extrapolate at 0.5 m (Indices 10 to 166) and interpolate at 2.0 m (Indices 312 to 468) and directly predicts the results at an observed altitude of 1.5 m (Indices 169 to 309).

Since each GPR’s estimate is a Gaussian distribution, we expected their estimation error to be within two standard deviations 95% of the time. By analyzing the red percentages in Figure 7, training on test_100 (top row) yielded more reliable error and uncertainty metrics than test_101 (middle row). As such, test_100 was used to train the indoor GPR-based magnetic field map. Once the accuracy of using GPRs to serve as maps of magnetic field was characterized, the maps were used to analyze the spatial variation of the magnetic fields in our indoor and outdoor test arenas.

### 5.2. Analyzing the Spatial Variation of Magnetic Fields

This section presents a new method to visualize 3D magnetic field maps and compares the magnetic field inside the Robotics Fly Lab to that of the outdoor M-Air facility. We show, qualitatively, that the magnetic field indoors has more spatial variation than outside.

**Figure 8:**
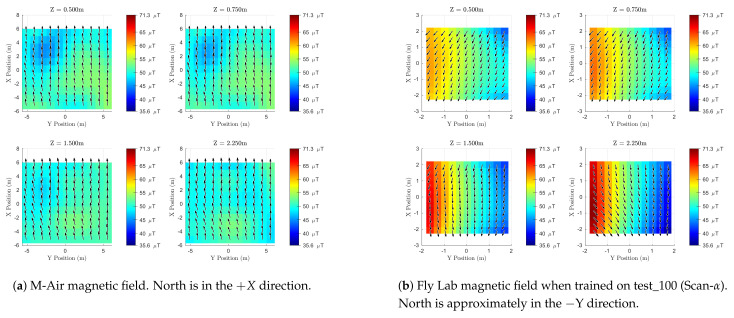
Outdoor (**a**) and indoor (**b**) magnetic field at four different altitudes. Black arrows show the direction of the planar (*X* − *Y*) component of the magnetic field. The color behind each arrow shows the strength and direction of the *Z* component of the magnetic field.

Figure 8a shows the GPR prediction of the magnetic field at the outdoor facility for four different altitudes. Black arrows indicate the horizontal field component, while the color behind each arrow depicts ${\tilde{B}}_{z}$ at that location. In Figure 8a, the +X direction is Geographic North. Figure 8b shows the GPR prediction for the indoor lab where Geographic North is approximately in the −Y direction. The outdoor magnetic field map (Figure 8a) was generated using 1157 observations of training data from the five outdoor flight profiles (Table 2), while the indoor map (Figure 8b) was generated using the 245 observations from test_100 (a Scan-α trajectory). The indoor map was qualitatively similar when trained on test_101.

At each location, the black arrow gives the local planar heading (or declination) of the magnetic field, which is equivalent to the direction a compass would point to at that location. To most people, this is the most familiar part of the magnetic field, and it is used to estimate heading. Parsing just the black arrows gives users an idea of how the local Magnetic “North” changes throughout a workspace and how reliable heading estimates might be without any magnetic field mapping or disturbance rejection. The color behind each arrow gives the strength and direction of ${\tilde{B}}_{z}$. This component of the magnetic field is often ignored if the magnetometer is used only for estimating heading. However, Figure 8b depicts features in ${\tilde{B}}_{z}$ that would be valuable for position localization, and in Section 5.5, we show how using ${\tilde{B}}_{z}$ can assist with roll and pitch estimates.

This method of visualization allowed us to analyze the relative strength of the ${\tilde{B}}_{x}$ and ${\tilde{B}}_{y}$ and the absolute strength of ${\tilde{B}}_{z}$. Unfortunately, these figures show no visual indication of how the horizontal (${\tilde{B}}_{x}$−${\tilde{B}}_{y}$) magnitude of the field compares to the vertical component (${\tilde{B}}_{z}$). For the indoor flight space, such comparisons can be made using Figure 7e,f. For the 1157 observations in the outdoor training dataset, the range of measured values for each component of the magnetic field was ${\tilde{B}}_{x}\in \left[15,\;24\right]\;\mu T$, ${\tilde{B}}_{y}\in \left[-7,\;2\right]\;\mu T$, and ${\tilde{B}}_{z}\in \left[48,\;54\right]\;\mu T$. Finally, there is no visual indication of the uncertainty of the GPR’s estimate in our figures. However, as in [12,24], one could use the transparency of the arrows and colors to indicate regions of lower certainty.

Returning to Figure 8a, note that the magnetic field at M-Air generally points towards Geographic North and into the ground. These characteristics remain fairly constant as a function of altitude. The total magnetic field strength varied from 51.6 μT to 57.6 μT for all points in the outdoor training dataset.

Figure 8b shows that the indoor magnetic field at 0.5 m was generally westward, but pointed more northwest as altitude increased. Recall that Geographic North is approximately in the −Y direction in this figure. Figure 7e,f show that ${\tilde{B}}_{z}$ is the largest component of the magnetic field indoors as well. The total magnetic field strength varied from 43.9 μT to 73.3 μT for all points in the indoor training dataset.

For the indoor magnetic field, ${\tilde{B}}_{z}$ had a larger range of values as altitude increased with both the smallest and largest values appearing at an altitude of 2.25 m. At all altitudes, ${\tilde{B}}_{z}$ had a larger magnitude on the −Y side and a smaller magnitude on the +Y side. The −Y wall is a movable divider between the Robot Fly Lab and an adjacent lab space. This divider has reconfigurable panels that run along a metal track about 3 m off the ground. We believe this metallic track and metallic portions of the panels were responsible for the strengthened *Z* field on the −Y side of the indoor flight lab.

The magnetic field of the indoor workspace had much more spatial variation than the magnetic field outside. The indoor field also changed noticeably as a function of altitude, a trend that was recently analyzed in [25]. In the next section, we show how high-resolution GPR-based magnetic field maps can be used for attitude estimation in regions with spatially varying magnetic fields.

### 5.3. Comparison of Magnetic Field References

This section shows that the high-resolution GPR-based magnetic field map is a better source of reference magnetic field vectors $r_{k,mag}$ when there is high spatial variation in the magnetic field. If the magnetic field has low spatial variation, the GPR’s reference vectors yield similar attitude estimate errors assuming the field is constant everywhere.

Table 4 shows the root-mean-squared error (RMSE) in degrees of the MEKF’s attitude estimator for all five flight profiles of the outdoor dataset. The “training” flight tests used to create the GPR-based magnetic field map were different from the “utilization” tests used here to evaluate attitude estimation error (Table 2).

For each flight test, the three table columns correspond to roll, pitch, and yaw RMSE in degrees. Estimated quaternions were converted to Euler angles using a 3-2-1 (yaw-pitch-roll) rotation sequence. The first column indicates the source of the magnetic field reference vectors. The first row labeled “Gyro/Accl Only” excludes magnetometer data; it uses only the gyro and accelerometer to perform attitude estimation. Yaw RMSE values were omitted here since heading is not observable with only gyro and accelerometer data. The second row is for WMM, which has such a low resolution that the reference vector is the same for all locations in the five flight profiles. The third row queries the GPR-based map at a single location and uses that single reference magnetic field vector regardless of the vehicle’s actual location. The position (0,0,−1.5) m was chosen since all flight profiles were centered around the origin and most had a fixed 1.5 m desired altitude. The exceptions are FP-D and FP-In-D, the inclined circle trajectories. This row can be interpreted as using the GPR with the same low-resolution WMM offers. Finally, the last row, labeled “GPR”, queries the GPR-based map at each time step to provide a specific magnetic field reference vector for each vehicle position to leverage the full interpolation capability of the GPR. We refer to this case as the “high-resolution” map and the other two, constant, reference vector methods as “low-resolution” alternatives. The bold value in each column corresponds to the reference vector source that yielded the lowest roll, pitch, or yaw RMSE.

Table 5 shows similar data, but now for the indoor dataset. Again, the “training” flight tests are different from the “utilization” tests used to evaluate the estimation error per Table 3. The format of Table 5 is the same as that described above. WMM’s magnetic reference vector is not representative of the magnetic field inside the Robot Fly Lab, so WMM was not considered for indoor analyses.

For each flight test, the same sensor data were played back to the MEKF with the only difference being the sensors available or the source of magnetic field reference vectors. The MEKF parameters were tuned by hand, resulting in reasonable, but not “optimal”, parameter values. The MEKF parameters were held constant across all indoor and outdoor flight datasets. Because of this, it is possible to have the overall lower RMSE values in Table 4 and Table 5 if the MEKF is tuned more specifically for the indoor or outdoor environment or specific flight profiles.

For the outdoor dataset in Table 4, the high-resolution map (“GPR” row) yields comparable attitude estimates relative to the low-resolution magnetic field reference sources. The high-resolution map tended to give lower (but comparable) roll error than the alternatives. Aside from FP-A and FP-C, the outdoor flight tests had noticeably higher yaw error in the “GPR” row. We believe this increased yaw error was caused from not calibrating the RM3100 magnetometer before performing the outdoor flight tests. Since the horizontal component of the magnetic field is much smaller than ${\tilde{B}}_{z}$ (Section 5.2), small errors due to lack of calibration would more easily change the measured heading of the magnetic field. This poses more of a problem to our yaw estimates than roll and pitch [4]. We would expect such small calibration errors to have less impact on yaw estimates in a more equatorial part of the world where the horizontal component of the magnetic field is more dominant.

Table 5 shows that when testing indoors, with more spatial variation in the field, the high-resolution GPR-based map consistently allowed the MEKF to achieve lower errors in roll, pitch, and yaw estimates. The most significant outlier was observed from test_119 where using the high-resolution map reduced the error in all the Euler angles by at least one-third. We believe this was because test_119 was a trial of FP-In-D (inclined circle) and spent more time in regions of the magnetic field that differed significantly than what was observed at the reference (0,0,−1.5) m state (see Figure 8b). In fact, similar trends occurred for test_115 and FP-In-A, the level rectangle that traced the perimeter of the flight space. In such cases, the constant ambient magnetic field assumption was inconsistent with the magnetometer’s observations as the vehicle flew.

From Table 4, we see that when the magnetic field of a workspace had a low spatial variation, having a higher resolution map did not reduce the attitude estimation error in general, but also did not make attitude estimates generally worse. The exception was with yaw estimation: which was consistently worse when we constantly changed the magnetic reference vector as the vehicle moved. However, the relatively larger yaw error was likely due to a lack of magnetometer calibration for the outdoor flights in combination with a small horizontal magnetic field. In contrast, Table 5 shows a promising environment where the high resolution of the GPR-based maps can be utilized to improve vehicle attitude estimation. In short, high-resolution GPR-based magnetic field maps can significantly improve attitude estimation if there is high spatial variance, but do not perform noticeably better or worse than the low-resolution alternatives when there is low spatial field variation.

It is important to note that the outdoor magnetic field map was trained using the flight profiles, while the indoor map was trained using Scan-α. In the next section, we show that both methods of training yielded similar accuracy when validating on the flight profiles.

### 5.4. Comparison of GPR-Based Map Training Methods

This section analyzes how the locations of the observations used in the training set affected the accuracy of the GPR-based map by comparing the accuracy of a GPR trained on Scan-α trajectories against a GPR trained on indoor flight profiles. We show that training on Scan-α yielded mean absolute errors of 0.28 μT, 0.27 μT, 0.48 μT, while training on the five indoor flight profiles gave mean absolute errors of 0.30 μT, 0.28 μT, and 0.48 μT for $B_{x}$, $B_{y}$, and $B_{z}$, respectively, when validated on the indoor flight profiles. As such, the outdoor GPR-based map was sufficiently accurate for the analysis conducted in Section 5.3, and the conclusions drawn from Table 4 and Table 5 are sound despite differences in the GPR training methodologies. Note that these metrics exclude validating on FP-In-A due to a sensor misalignment during test_105 (FP-In-A training flight).

**Figure 9:**
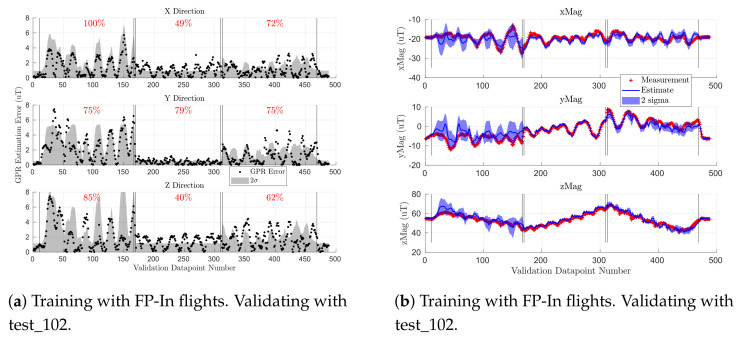
Accuracy of the indoor GPR-based magnetic field map when trained on the five indoor flight profiles (FP-In flights) and validated on test_102 (Scan-*β*). The large regions separated by the black vertical lines show areas of constant desired altitude for the Scan-*β* trajectory.

Figure 9 shows the accuracy of the indoor magnetic field map when trained on five indoor flight profiles (FP-In) and validated on a Scan-β flight. The left plot is the GPR estimation error when validated on flight test_102, while the right plot shows the GPR estimates (blue line) against observations from the validation set (red cross). The results were qualitatively similar when validating on test_103. The blue and gray shaded regions depict two standard deviations of GPR uncertainty at each sampled location. The black vertical lines partition the figure into sections of constant altitude (0.5 m, 1.5 m, and 2 m, respectively). We used samples from test_105, test_107, test_110, test_112, and test_114 as shown in Table 3 to train the GPR on the FP-In trajectories. Figure 7 shows similar metrics for the indoor GPR-based map when trained on the lawnmower coverage pattern Scan-α.

Figure 9 shows that the error on the 1.5 m sweep was much lower than for the other two altitudes. This is because most of the flight profiles in the FP-In training set were trajectories at an altitude of 1.5 m. As such, the GPR trained on FP-In had a good understanding of the magnetic field at this altitude, but not at 0.5 m and 2.0 m, the other Scan-β altitudes. This was expected since Gaussian processes tend to only provide accurate predictions near data found in their training set.

Above, we showed that when validating on Scan-β, training on Scan-α (Figure 7) yielded better results than training on FP-In trajectories (Figure 9). However, the MEKF did not use data from the Scan-β trajectories, so it was important to analyze the accuracy of the two mapping methodologies on FP-In flights as well.

**Figure 10:**
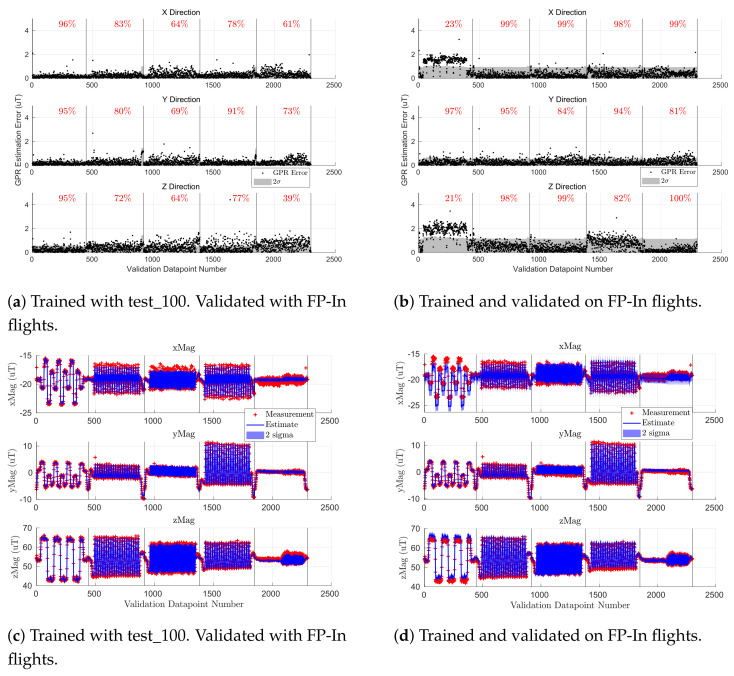
The accuracy of the indoor GPR-based magnetic field map when *validated* on the five indoor flight profiles (FP-In flights). The black vertical lines separate each of the five indoor flight profiles. The steady-state error in Figure 10b was likely caused by a misalignment with the magnetometer in test_105 (FP-In-A training flight).

Figure 10 shows the results of validation with the five FP-In validation flights (test_115, test_116, etc.; see Table 3). The left figure column shows the results when training on Scan-α, and the right column was trained on the five FP-In training flights. Here, in contrast to other similar plots, the vertical black lines separate each of the five FP-In validation flights (in order of FP-In-A to FP-In-E). We can see that when validating on the intended flight profiles, both training methodologies had a similar error. The exception was the steady-state error of $B_{x}$ and $B_{z}$ when validating on the level rectangle (test_115 of FP-In-A) in Figure 10b,d.

Figure 10d shows that the steady-state shift for FP-In-A was consistent with the magnetometer being pitched slightly in the training flight (blue line) relative to the validation flight (red cross). Although there were no significant crashes during the indoor flight tests, there were some rough landings that occasionally dislodged some standoffs and screws. It is possible such a harsh landing misaligned the RM3100 magnetometer relative to the rest of the rigid body. Since we only saved the data from the successful flights, this misalignment must have happened on an unsuccessful flight just before test_105, but was fixed before test_107. Note: this hypothesis also explains the sinusoidal $B_{x}$ and $B_{z}$ error trends on the 1.5 m altitude section of Figure 9a where the peaks in errors occurred when the Scan-β trajectory was on the outer rectangular perimeter of the flight space (i.e., overlapping most with test_105), and the dips occurred when Scan-β passed through the center region with the data from the flight tests that had a properly aligned magnetometer. Excluding FP-In-A, training on Scan-α yielded mean absolute errors of 0.28 μT, 0.27 μT, 0.48 μT while training on the five indoor flight profiles gave mean absolute errors of 0.30 μT, 0.28 μT, and 0.48 μT for $B_{x}$, $B_{y}$, and $B_{z}$, respectively.

As such, Figure 10 shows us that the two training methodologies yielded comparable results when validating on the FP-In flight profiles and highlights the importance of accurate IMU sensor alignment for every flight. Due to the issue with test_105 (FP-In-A) and that querying the GPR was faster when it was trained on fewer data, we used test_100 (Scan-α trajectory) to train the indoor GPR during the analysis of Section 5.3. However, we showed that training the indoor GPR on the five indoor flight profiles would likely yield similar results to Table 5.

This brings us to an important point. If the intended route through an indoor workspace is known a priori, then it is sufficient to construct a magnetic field map by gathering observations along the path. This kind of mapping methodology is common with self-driving cars where maps are only generated along roads the vehicle intends to traverse. However, training magnetic field maps from coverage patterns such as Scan-α will generally yield more accurate results throughout the workspace.

In summary, we used the indoor flight space to compare two mapping methodologies and showed that mapping with the Scan-α coverage pattern yielded a uniformly more accurate map when validating on Scan-β, but had comparable accuracy when validating on the indoor flight trajectories. Since the outdoor GPR was trained using the five outdoor flight profiles, we can conclude that our outdoor map is sufficiently accurate near the locations of the five flight profiles.

### 5.5. Magnetometer Correction of Accelerometer Roll/Pitch Errors

This section shows that magnetometers can resolve accelerometer roll/pitch errors observed during maneuvers. For this analysis, we focused on test_117, a FP-In-C trajectory from the indoor dataset. Recall that all FP-In-C flights followed a level circle with a radius of 1m, a commanded tangential velocity of 1.5 m/s, and a commanded heading that changed to keep the +X body axis tangent with the circle. As such, in an ideal flight, the vehicle would experience 2.25 m/s${}^{2}$ of centripetal acceleration along its *Y* axis.

**Figure 11:**
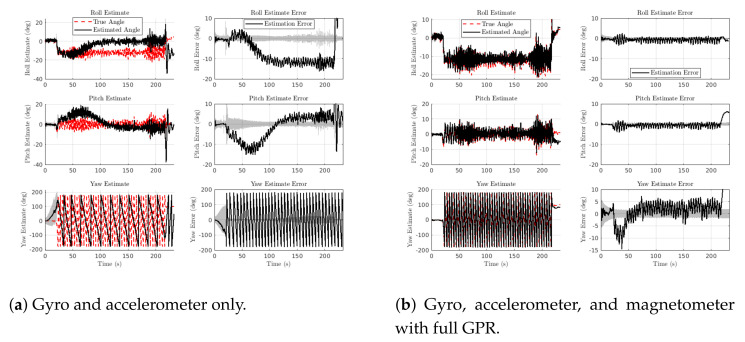
Time series plots for roll, pitch, and yaw estimates of test_117 (fast level circle). Linear and centripetal accelerations caused pitch and roll errors (respectively) in Figure 11a that were corrected with the magnetometer in Figure 11b.

Figure 11a shows the roll, pitch, and yaw estimates along with their respective errors when using only the gyroscope and accelerometer. A 20 s into the flight, the vehicle accelerated from rest to achieve the 1.5 m/s desired tangential velocity. Acceleration was measured primarily in the body *X* direction, which causes a transient MEKF pitch error around the 65 s time mark. As the vehicle reached its maximum speed, the accelerometer measured centripetal acceleration in the body *Y*-direction, causing a steady-state roll error over most of the flight.

This is a common problem for flight vehicles [45,46,47] and other platforms [7,8,9,10] that use the accelerometers for attitude estimation. Typically, the accelerometer’s measurement is discarded or de-emphasized when nongravitational accelerations or sensor anomalies are detected. Here, we circumvented the need to detect nongravitational accelerations by simply allowing the magnetometer to assist with roll and pitch estimation.

Figure 11b is the same flight, but now with the magnetometer. Here, we see that both the transient pitch error and the steady-state roll error were eliminated, demonstrating that the information from the magnetometer and the GPR-based map was able to improve roll and pitch estimates. This is important because, in many applications, the magnetic field is only used to provide yaw (heading) measurements, while roll and pitch are left to the accelerometer. However, with sufficient information of the magnetic field, magnetometers can also assist in correcting roll and pitch estimates.

It is important to qualify when magnetometer adjustment of roll and pitch estimates will be beneficial. Table 5 shows that for FP-In-C, roll and pitch estimates were improved even with a constant (low-res) magnetic field reference at all points. This improvement increased when using the high-resolution, full GPR. FP-In-E showed a similar trend since the magnetometer was able to offset errors in the attitude estimates caused by the acceleration of maneuvering. However, with FP-In-A, where the vehicle was hovering for much of the flight, the magnetometer with a low-res GPR map actually made roll and pitch estimates worse. This is because the indoor flight space had spatial variation in the magnetic field that was not captured by assuming a constant reference source. However, with the full GPR, we again see the magnetometer able to improve roll and pitch estimates past initial accelerometer estimates. Finally, such roll and pitch corrections may not be viable in equatorial parts of the planet where the the horizontal component of the magnetic field is dominant.

## 6. Conclusions and Future Work

This paper presented methodologies to train, validate, and visualize 3D GPR-based magnetic field maps and examined their value for indoor attitude estimation. We presented a new visualization technique to better understand the spatial variation of 3D magnetic fields and discussed its advantages and drawbacks. Our visualization technique relies on some interpolative tool, such as GPR, to estimate the magnetic field at locations not found in the training set. Next, we showed that in spaces with high spatial variation, high-resolution GPR maps can improve attitude estimates, but these maps did not yield significantly better or worse attitude estimates in spaces with low spatial variation. Finally, we demonstrated two ways of training GPRs and showed that training a GPR-based map using coverage flight trajectories such as Scan-α was generally better, though constructing a map using only observations along flight routes of interest was just as accurate in the vicinity of the flight route. We also provided an illustrative example of how magnetometers can assist with roll and pitch estimates in a workspace with high magnetic spatial variation.

In future work, it will be critical to better understand how much spatial variation an environment needs before it becomes advantageous or necessary to use magnetic field maps of the workspace. We plan to apply quantitative methods of describing the spatial variation of a workspace and investigate how workspaces with spatially varying magnetic fields can be used to improve position estimates. The goal is to perform full 6DOF position and attitude estimation using GPR-based magnetic field maps [12]. This would require an analysis of multiple indoor spaces as in [4,25], which is currently difficult due to our dependence on motion capture cameras to construct our maps. Previous works have found success with using LiDAR or visual–inertial odometry to construct magnetic field maps without motion capture [12,15,16,20,25]. These methods are recommended to facilitate accurate magnetic field mapping in complex regions where nearby structures offer distinct mapping information for LiDAR and vision sensors. Furthermore, we aim to investigate how to better address the time-varying nature of magnetic fields [24] to more accurately represent the magnetic field near large moving ferromagnetic structures such as elevators and doors [11,19,24].

## Figures and Tables

**Table 1:** Calibration parameters of RM3100 magnetometer from indoor flight tests.

Scale Factor (−)			Bias (μT)			Non-Orthogonalities (°)		
a	b	c	$x_{0}$	$y_{0}$	$z_{0}$	ρ	λ	ϕ
1.01	0.955	0.942	−1.26	−2.46	3.24	0.182	2.28	−0.118

**Table 2:** Outdoor flight profiles from Section 4.2 were split into a “training” set (to create the GPR map) and a “utilization” set (to analyze attitude estimation). Each flight profile contributes a single training flight and a single utilization flight.

	Training (1.0 Hz)	Validation	Utilization (Max Sensor Frequency)
FP-A	test_01	–	test_10
FP-B	test_03	–	test_11
FP-C	test_05	–	test_12
FP-D	test_07	–	test_13
FP-E	test_09	–	test_14

**Table 3:** The indoor flight profiles from Section 4.3 have dedicated flight trajectories for GPR training and validation. The indoor experimental profiles match the outdoor versions, but with smaller footprints to fit in the spatially constrained indoor motion capture arena.

	Training (1.0 Hz)	Validation (2.0 Hz)	Utilization (Max Sensor Frequency)
Scan-α	test_100 & test_101	–	–
Scan-β	–	test_102 & test_103	–
FP-In-A	test_105 (2Hz)	test_115	test_115
FP-In-B	test_107	test_116	test_116
FP-In-C	test_110	test_117	test_117
FP-In-D	test_112	test_119	test_119
FP-In-E	test_114	test_120	test_120

**Table 4:** RMSE for each *outdoor* experimental test using different magnetic field reference sources. The first row excludes magnetometer data. For each test, the three columns show the RMSE for roll, pitch, and yaw, respectively, in degrees.

*RMSE* (${}^{\circ }$)	FP-A			FP-B			FP-C			FP-D			FP-E		
*Roll|Pitch|Yaw*	test_10			test_11			test_12			test_13			test_14		
Gyro/Accl Only	**1.6**	2.2	–	2.6	2.0	–	10	4.2	–	3.2	2.2	–	7.8	10	–
WMM	**1.6**	0.7	**2.3**	2.3	**1.5**	**2.2**	3.2	**1.4**	**4.8**	2.5	**1.4**	**2.2**	**1.8**	0.7	**1.9**
GPR (0, 0, −1.5) m	1.7	**0.4**	2.6	2.5	1.7	2.4	3.4	1.6	5.1	2.7	1.6	2.5	1.9	**0.4**	2.2
GPR	1.7	0.7	2.7	**1.7**	1.9	4.5	**2.2**	1.8	5.5	**1.3**	2.5	3.7	**1.8**	0.5	4.7

**Table 5:** RMSE for each *indoor* experimental test using different magnetic field reference sources. The first row excludes magnetometer data. For each test, the three columns show the RMSE for roll, pitch, and yaw, respectively, in degrees.

*RMSE* (${}^{\circ }$)	FP-In-A			FP-In-B			FP-In-C			FP-In-D			FP-In-E		
*Roll|Pitch|Yaw*	test_115			test_116			test_117			test_119			test_120		
Gyro/Accl Only	1.1	0.8	–	2.5	1.5	–	10	6.7	–	2.2	1.6	–	1.5	1.3	–
GPR (0, 0, −1.5) m	2.6	4.7	9.0	2.5	3.2	11	2.9	3.6	13	4.9	3.8	11	1.1	0.6	5.0
GPR	**0.7**	**0.2**	**1.7**	**1.1**	**0.9**	**3.7**	**1.1**	**1.7**	**5.0**	**0.8**	**0.9**	**2.7**	**1.0**	**0.4**	**1.9**

## Data Availability

Not applicable.
